# Maritime urban tracking dataset in harbor environment

**DOI:** 10.1038/s41597-026-07488-6

**Published:** 2026-06-02

**Authors:** Nicholas Dalhaug, Trym Anthonsen Nygård, Miguel Hinostroza, Henrik Dobbe Flemmen, Emil Martens, Annette Stahl, Rudolf Mester, Edmund Førland Brekke

**Affiliations:** 1 Department of Engineering Cybernetics, the Norwegian University of Science and Technology (NTNU), Trondheim, Norway; 2 Department of Computer Science, NTNU, Trondheim, Norway

**Keywords:** Computer science, Information technology, Software

## Abstract

Maritime target tracking datasets are crucial for developing and benchmarking algorithms that support safe navigation of intelligent marine vessels in congested environments. Unlike the automotive domain, where benchmarks like KITTI have driven progress, maritime datasets with ground truth remain limited. This paper introduces the Maritime Urban Tracking (MUT) dataset for perception and tracking in urban waters for autonomous surface vessels. Data were collected using an autonomous ferry prototype for stereo matching, optical flow, Simultaneous Localization and Mapping (SLAM), 2D/3D object detection, water segmentation, and tracking. The ego-vessel is equipped with short- and wide-baseline stereo cameras, Light Detection And Ranging (LiDAR), Realt-Time Kinematic (RTK) Global Navigation Satellite System (GNSS), Inertial Measurement Unit (IMU), and a polarized stereo rig. Some targets feature dual GNSS receivers with Post-Processed Kinematics (PPK) for accurate world-frame reference tracks. The dataset includes 19 tracking scenarios, 8 calibration sequences, 1 mapping scenario, and 3 docking scenarios, mostly 1 minute long, recorded at 30 fps (cameras) and 10 Hz (Lidar). Public release aims to lower entry barriers and foster innovation.

## Background & Summary

Maritime target tracking has typically employed ranging sensors such as Light Detection And Ranging (LiDAR) and Radio Detection And Ranging (RADAR). When vessels are far away and well-separated from land, a RADAR can be used to detect the vessel because of its long range. For closer distances, the Lidar gives more precise localization of targets, denser measurements and greater vertical resolution than the typical maritime RADARs. For tracking using these sensors it is useful with reference tracks in the world frame, for example from Global Navigation Satellite System (GNSS) receivers with Post-Processed Kinematics (PPK) for precise reference localization.

However, maritime urban tracking often involves targets in close proximity to each other and to land. In such scenarios, the relatively low spatial resolution of typical RADARs can pose significant challenges. A Lidar might still be very useful, but it can have difficulties with differentiating between different targets or between a target and land. This is where cameras can be advantageous. They have a higher density of measurements per frame, although typically with reduced field of view. Their measurements are also rich with information as each pixel contains multiple color channels which gives every image abundant contextual information that is typically extracted through deep learning methods.

Visual target tracking is a popular field with benchmarks such as the Visual Object Tracking (VOT) challenge^1^ or the Multiple Object Tracking (MOT) challenge^2^. These benchmarks use monocular images and track objects in the image frame. Datasets for monocular visual tracking in the maritime domain include the Singapore Maritime Dataset^3^ which is in a persons point-of-view and the SeaDronesSee benchmark^4^ which is in a drone’s point-of-view. The last example was used in the Maritime Computer Vision (MaCVi) challenge^5^ for comparing target tracking methods. It is useful to have 2D object detection datasets^6–10^ and image tracking datasets also for 3D tracking^11^. However, none of these datasets allow for comparison with world frame reference tracks.

While there are maritime datasets for RADAR data^12^, a dataset that uses a stereo camera is the USVInland dataset^13^. It focuses mainly on visual artifacts, stereo matching, and image segmentation. It uses a short baseline stereo camera only and does not focus on target tracking. The most relevant existing dataset is the PoLaRIS dataset^14^ which is based on the Pohang Canal dataset^15^ and includes multiple LiDAR sensors and cameras. The images and LiDAR scans are labeled to provide world frame reference tracks. However, these tracks are derived from the Lidar data rather than from independent measurements, e.g., GNSS. Furthermore, the moving targets are generally not located near land and are not on a collision course with the ego-vessel. The MIT Sea Grant Marine Perception Dataset^16^ has GNSS receivers on some targets. However, these do not appear to use PPK, and the targets are located far from land across a wide canal. Excluding simulation-based datasets^17,18^, there does not appear to be a visual dataset that combines stereo cameras with PPK GNSS world frame reference tracks. For an overview of maritime visual datasets see Su *et al*.^19^

There are benchmarks in the automotive domain that focus on target tracking, such as KITTI^20^, nuScenes^21^, and Waymo^22^. These benchmarks have laid the foundation for major developments within autonomous systems. For example, the field of scene flow estimation has progressed following the addition of scene flow ground truth^23^, and synthetic benchmarks have subsequently emerged^24–27^. We would like to see similar developments within the maritime domain.

In this dataset, we combine visual target tracking with classical target tracking using stereo cameras and Lidar for 3D target tracking with reference tracks from GNSS sensors. In addition, we provided detailed descriptions of the equipment calibration process for improvements in future research. We therefore add the calibration sequences to the dataset. Earlier versions of our dataset have been used in multiple publications on target tracking^11,28–31^, scene representation^32,33^, scene flow^34^, and water surface estimation^35^. By publishing this dataset, we enable more researchers world-wide to contribute to maritime autonomy without requiring an autonomous platform.

Our main contributions are as follows:

- We release a dataset for maritime visual and Lidar-based target tracking in urban waters that includes
  - Short and wide baseline stereo camera configurations in a 6-camera setup
  - A polarized stereo camera
  - Scenarios focusing on targets close to land
  - PPK GNSS reference tracks
  - Dual target GNSS receivers for heading reference independent of other sensors
- We provide a detailed description of the sensor setup and calibration procedures.

The paper is organized as follows: First we describe the methods and sensors used to gather the dataset, including synchronization and calibration. Second, we explain how the data is stored in the Data Records section. Third, we give an overview of the content of the dataset, including explaining the environment and scenarios. Fourth, we show technical validation of the dataset, and describe where to get the data as well as code to interface the data. Finally, we have added figures and description of all scenarios in Figs. 10,11 and Table 6.

## Methods

This section describes the methods and sensors used to record the dataset, including methods of synchronization and calibration.

### Ferry Prototype Sensor Platform

**Fig. 1:**
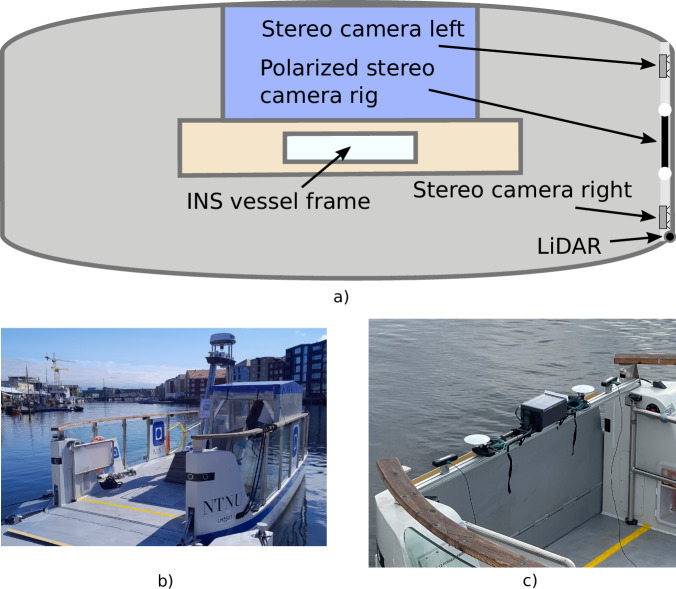
Ferry overview. (**a**) Drawn illustration of the ferry with the dataset sensors. (**b**) Photo of ferry. (**c**) Photo of sensors.

The ferry prototype used for data gathering is milliAmpere2^36^. The ferry is equipped with an onboard navigation system. For this dataset, we add additional sensors, listed in Table 1. These sensors are grouped as shown in Fig. 1. Although the RADAR is visible on the top of the ferry, it is not included in the dataset.

**Table 1:** The sensors used on the autonomous ferry prototype.

Sensor	Description
INS/GNSS	An early SentiSystems Inertial Navigation System (INS)^37^, but with an ADIS16495 Inertial Measurement Unit (IMU). The system uses two Realt-Time Kinematic (RTK) GNSS antennas and uBlox F9P receiver board. Operates at 100 Hz.
LiDAR	Ouster OS1 32 Lidar, operating at 10 Hz.
Stereo cameras	Two ZED X cameras connected to a ZED Box. Operate at 30 Hz with a resolution of 1080 by 1920 pixels for each of the 4 monocular cameras. Compressed with H.265.
Polarization cameras	Two Triton 5.0 MP Polarization Model IMX264MYR cameras. Configured to operate at 12 Hz, capturing 12-bit raw data video with a resolution of 2048 by 2448 pixels.
GNSS tracks	Uses dual-band antennas and an uBlox F9P for each antenna. Data stored to SD-card at 5 Hz. Post-processed, PPK, using RTKLIB^38^.

#### Inertial Navigation System

The INS/GNSS, provided by SentiSystems, integrates a dual RTK GNSS and an IMU. This system outputs the ferry’s ego-pose at a frequency of 100Hz. The ego-pose is described in relation to a specific reference frame in the world, with the INS/GNSS reference frame named “vessel” and the world reference frame named “piren” in the dataset.

#### LiDAR

The Lidar is an Ouster OS1 32-beam. It provides a vertical resolution of 32 beams and a horizontal resolution of 2048 beams, operating at 10Hz. The sensor also includes an internal IMU, which can be used for calibration. Due to its mounting configuration, the full field of view is not available and is limited to slightly more than 180°.

#### Stereo Cameras

We use two commercially available ZED X stereo cameras from StereoLabs, each connected to a ZED Box. Each camera records at 30fps with a resolution of 1920 by 1080. The cameras are equipped with global shutters and polarizing lenses. The pixel size is 3 *μ*m ^39^ and the focal length is approximately 2.2 mm. This focal length changes a bit between the sequences, but they are static for each sequence. This seems to be caused by auto-focus from the stereo cameras. Note, to achieve real-time recording at 30fps the dataset is compressed using H.265 compression.

The stereo cameras are mounted approximately 2 m apart, with an internal baseline of about 12 cm each. This configuration provides both a short baseline and a wide baseline stereo setup, which is relatively uncommon but has interesting properties^28^. The cameras have been mounted on an aluminum beam and angled slightly upward and toward each other. The rigid mounting ensures that the sensors remain stable as the ferry maneuvers.

#### Polarization Cameras

**Fig. 2:**
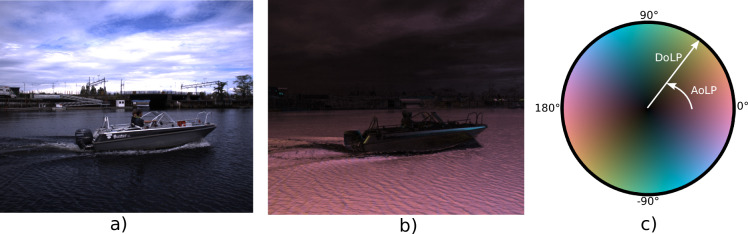
The polarization image uses a colormap to illustrate Degree of Linear Polarization (DoLP) and Angle of Linear Polarization (AoLP). The clear contrast in polarization between the water surface and rest of the scene highlights the potential usefulness of this data in the maritime domain. Further details are provided in Martens *et al*.^40^ (**a**) Color image. (**b**) Polarized image. (**c**) Colormap.

We used a sensor rig with two polarization cameras to capture stereo polarization video. The cameras are configured to operate at 12 Hz, capturing 12-bit raw data video at a resolution of 2048 by 2448 pixels^40^. In addition to color, these cameras capture information about the polarization state of light in the scene, which can be represented in terms of degree and angle of polarization^40^, DoLP and AoLP, as shown in Fig. 2. As reflections on the water surface have a distinct polarization signature, polarization cameras may be particularly useful for target tracking and other applications in the maritime domain, where reflections are typically considered visual artifacts^40^.

#### GNSS tracks

The main targets in this dataset are equipped with GNSS receivers configured for PPK. During data collection, raw GNSS measurements are recorded. These measurements are subsequently processed using RTKLIB^38^, together with observational data from a nearby base station, to generate accurate reference tracks. The base station is at Byåsen in Trondheim and is supplied by the Norwegian Mapping Authority. The settings used in RTKLIB are the default ones, filtering forward and backward again. The generation of reference tracks, including use of two antennas for getting heading, is described in Dalhaug *et al*.^31^, while the placement of the antennas are shown in Fig. 9, in the Calibration section under Data Overview.

### Synchronization

All sensors share a common time reference, specifically GNSS-time/Coordinated Universal Time (UTC)-time based on GNSS. However, this does not imply that they are triggered simultaneously, as the sensors operate on different computers without direct connections. For example, when some camera images are recorded at three times the frequency of the Lidar, the closest camera image in time is used for data association.

#### Stereo Camera Synchronization

The ZED stereo cameras are synchronized by the ZED Box, which ensures that the two stereo cameras are triggered almost simultaneously. To synchronize the box with the rest of the sensors, its internal clock has to be aligned with UTC-time. The box containes a GNSS receiver, but the antenna is not connected to the Pulse Per Second (PPS)-pin, preventing direct use of GNSS as an accurate timing source. Furthermore, the box lacks Precision Time Protocol (PTP) support, meaning hardware designed to synchronize timing on the network-card. Therefore, we use an Network Time Protocol (NTP) server with minimal latency, implemented on a Raspberry-Pi (RPi) connected directly to the box with a short ethernet cable. The RPi itself is connected to a GNSS receiver with the PPS signal enabled. The configuration results in a timing error of approximately 91ns on the RPi and about 121*μ*s on the box, according to measurements from the “chrony” software. Such low error values when using NTP are likely due to the short cable and the direct communication between the RPi and the box.

#### LiDAR and INS/GNSS Synchronization

The Lidar and INS/GNSS are both recorded on the ferry’s computer, which also is synchronized to UTC-time using GNSS from the INS/GNSS. These measurements are recorded using Robot Operating System (ROS).

#### Polarized Stereo Rig Synchronization

The sensor rig operates as an independent unit with its own computer, synchronized separately to UTC. Synchronization is achieved by using the PPS signal from one of its GNSS receivers to synchronize the computer clock to UTC, and PTP to synchronize the cameras with the computer^40^. The sensor rig captures and stamps 12 stereo frames per second, starting every whole second, with an offset error between the stereo images less than 20 *μ*s and an offset error relative to UTC less than 100 *μ*s ^40^. The accuracy was verified by comparing the trigger output of each camera with the PPS signal using an oscilloscope^40^.

### Participation Consent

Some participants of the data acquisition are identifiable in some images. They have given their consent to the publication of the data and the acquisition is approved by NTNU and SIKT, Norway, reference number 152466. A form with the formal information of what the data is for and how it is processed was sent to all participants and was accepted by all.

### Calibration

Correct sensor calibration ensures that all the relevant physical properties of each sensor (intrinsic parameters) and between the sensors (extrinsic parameters) are known. This is important for data processing and sensor fusion.

**Fig. 3:**
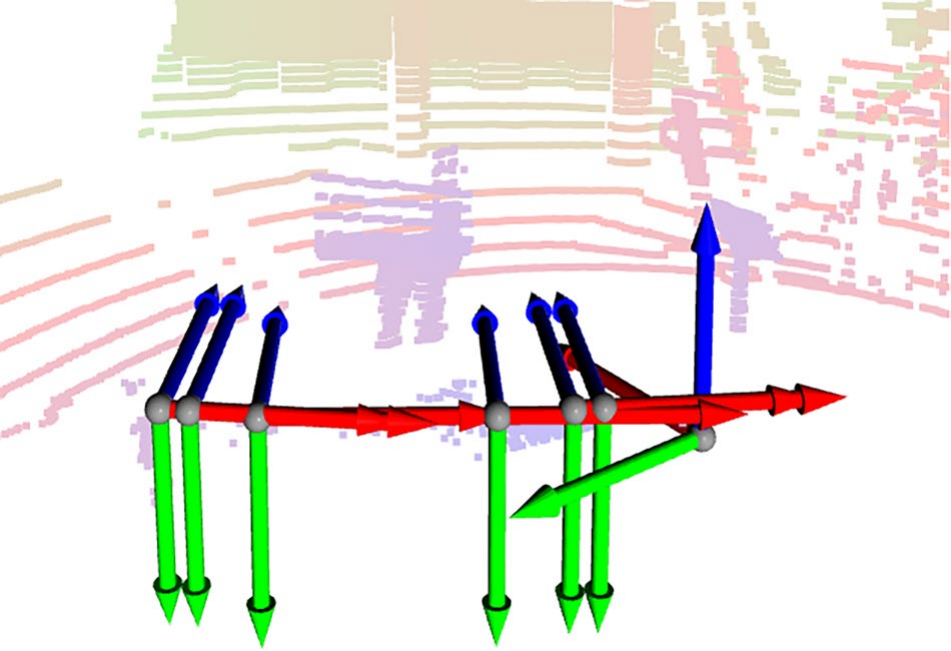
The resulting reference frames after calibration are shown here for the two stereo cameras, the polarized rig and the Lidar. See Fig. 1 for what reference frame corresponds to what sensor. The cameras are pointing towards the person with the calibration board, similarly to Fig. 4. The Lidar points are brightly colored based on distance to visualize the 3D structure. The x-axis is red, the y-axis is green and the z-axis is blue.

Figure 3 illustrates the relative positions of sensors, visualized from left to right: two cameras (short baseline stereo camera), the left camera on the polarized rig, the right camera on the polarized rig, two cameras (short baseline stereo camera) and the Lidar. More details are shown in the Technical Validation section.

#### Stereo Camera Calibration

**Fig. 4:**
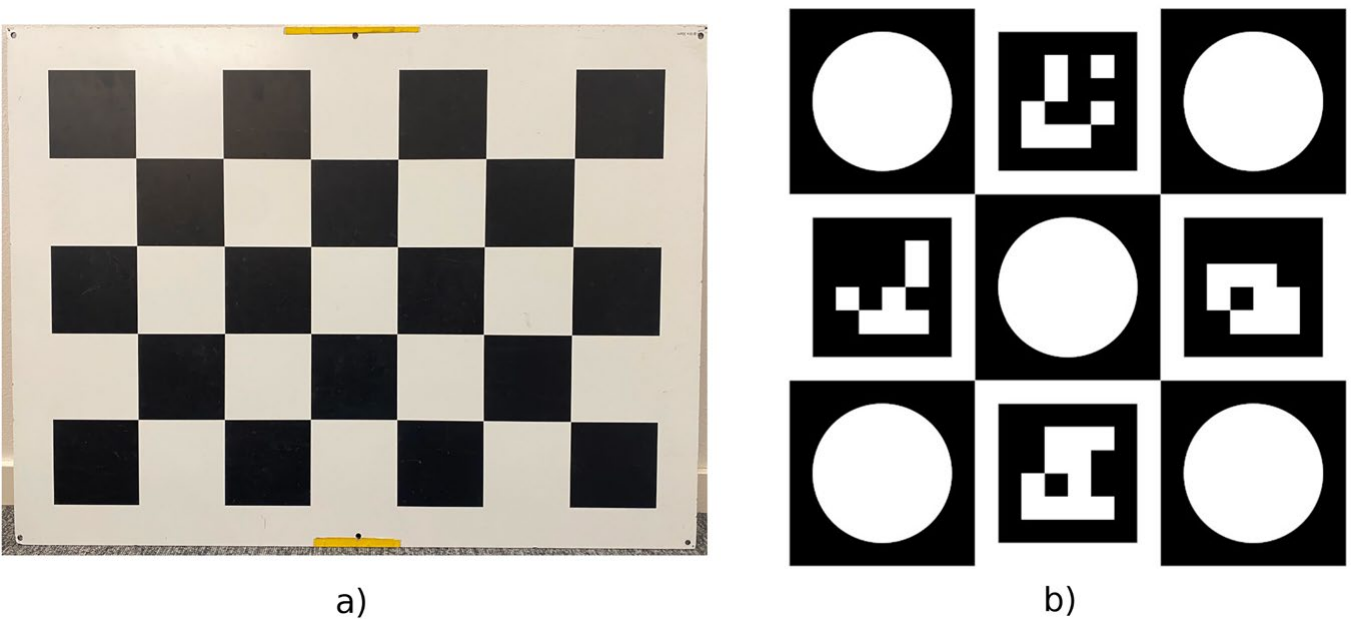
The calibration boards used in this dataset. (**a**) Photo of the stereo camera calibration board that was used. Each black square has a side length of 10.1 cm. (**b**) The calibration board used for extrinsic calibration between the Lidar and a camera.

Each of the two independent stereo cameras have their extrinsic and intrinsic parameters calibrated from the manufacturer. However, we calibrate the stereo cameras with respect to each other. Those extrinsic parameters correspond to the relative transformation between the two stereo cameras, the rotation and translation. This transformation is estimated using the calibration board shown in Fig. 4. For each of the two data collection days, we record two sequences dedicated to stereo calibration. We only use images we regard as sharp enough for the calibration.

#### LiDAR to Camera Calibration

In order to achieve a good initial transformation between the Lidar and one of the cameras, we use the calibration board shown in Fig. 4. A similar concept was applied in Yan *et al*.^41^ and Cholakkal *et al*.^42^ The software used to print the image onto an aluminum plate required 1.1 cm along the border. The image is 12000 px wide and high, with a resolution of 12 px/mm, making the board width and height 97.8 cm. The four makers are 4 × 4 ArUco markers^43^. Each of the nine squares on the board have the width and length of 8 smaller squares, where each smaller square has the width and height of 444 px. The circles have 6 such small squares in diameter, making the diameter $6\cdot 444\,{\rm{px}}/\left(12\frac{{\rm{px}}}{{\rm{mm}}}\right)=222\,{\rm{mm}}$. These circles are cut out, making holes that are detectable with the Lidar.

The estimation of the transformation starts with detecting the holes in each Lidar frame and detecting the ArUco markers in the corresponding image frame. Then we estimate the plane that the calibration board lies in using the Lidar data, and fit our model of the calibration board to the lidar data in a 2D Iterative Closest Point (ICP) optimization step. From this fit, we extrapolate the positions of the ArUco markers within the Lidar point cloud, enabling marker detection in both the image and the 3D Lidar data. Finally, we apply a Perspective-n-Point (PnP) RANdom SAmple Consensus (RANSAC) solver^44^ to compute the transformation between the Lidar and camera.

**Fig. 5:**
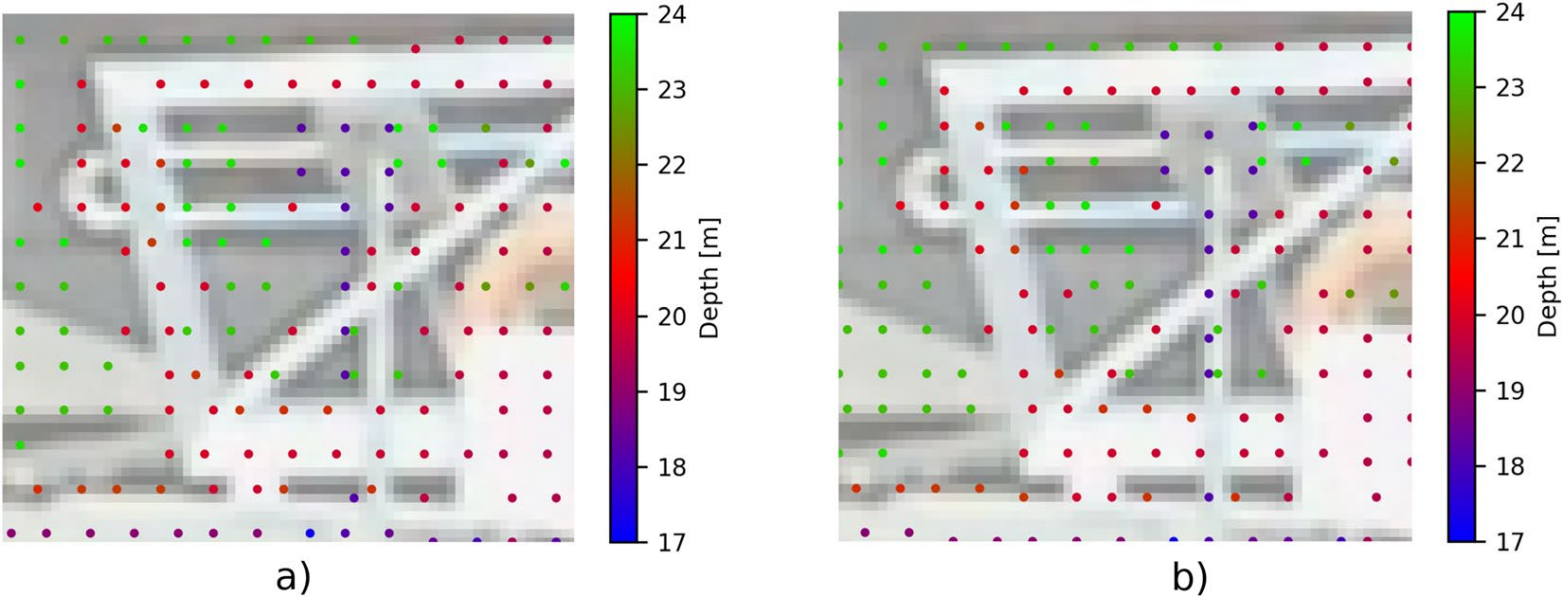
Lidar points projected into the image before and after manual tuning. Point colors represent depth, while the corresponding image is brightened and shown in the background. (**a**) Before manual tuning. (**b**) After manual tuning.

However, the resulting transformation did not appear accurate enough. This is likely due to the sparsity of the Lidar point cloud, as the sensor provides only 32 vertical beams. The issue becomes particularly severe at larger distances, tens of meters, where the holes on the calibration board are no longer visible, leading to inaccuracies in the rotation estimate. As illustrated in Fig. 5, the purple Lidar points on the tablet and pole have been shifted to the right to achieve better alignment with the image, shown here at a distance of approximately 20 m.

#### LiDAR to INS Calibration

We find the transformation from the Lidar to INS/GNSS by aligning the trajectory we get from Lidar inertial odometry(LIO) to the trajectory from the INS. This is equivalent to the traditional hand-eye calibration problem in robotics, where we minimize:

1 $$\mathop{\min }\limits_{{T}_{g}^{l},{T}_{{w}_{l}}^{{w}_{g}}}\mathop{\sum }\limits_{k=0}^{N-1}{\left\Vert {\rm{Log}}{\left({T}_{{w}_{l}}^{l}[k]\right)}^{-1}\,\circ {T}_{g}^{l}\,\circ {T}_{{w}_{g}}^{g}[k]\,\circ {T}_{{w}_{l}}^{{w}_{g}}\right\Vert }_{\Sigma }^{2}$$

where

${T}_{b}^{a}$: is the rigid body transformation relating frame *a* and *b* such that ${p}^{a}={T}_{b}^{a}* {p}^{b}$ for a point *p*^*b*^ in frame *b*.

${T}_{b}^{a}[k]$: is the rigsid body transformation relating frame *a* and *b* at timestep *k*.

*N*: is the number of timesteps in the optimization.

∘: is the *SE*(3) composition operation^45^.

*l*: is the Lidar frame.

*g*: is the INS/GNSS frame.

*w*_*l*_:is the world frame for the Lidar.

*w*_*g*_: is the world frame for the INS.

*Σ*: is a weighting matrix between the orientation and position component of the pose.

${\rm{Log}}T$: is the vector with the elements of the tangent space representation of *T*^45^.

$\parallel \cdot {\parallel }_{\Sigma }^{2}$ is the Mahalanobis distance weighted with *Σ*.

We use the state-of-the-art algorithm Fast-LIO2^46^ with the internal IMU in the Lidar. The transformation from the IMU frame to the Lidar frame is given in the documentation.

The INS/GNSS samples the pose at a 100 Hz while the LIO operates at 10 Hz. The pose sampling is not synchronized, but they are both captured relative to the same clock. Thus we select the trajectories for the optimization by taking every pose from the LIO and associating it with the nearest (in time) pose from the INS/GNSS.

**Fig. 6:**
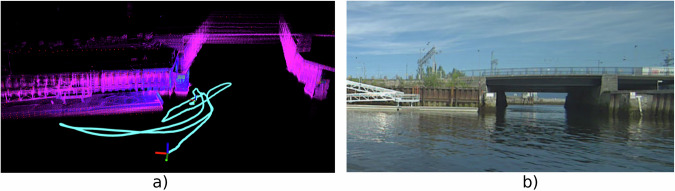
The path we made (in cyan) for calibrating the Lidar to the INS/GNSS. The purple background are the accumulated Lidar scans from Fast-LIO2^46^. We can see the bridge to the right and the dock to the left in the image. Black parts do not have Lidar points so they are either water or simply parts of the scene that has not been observed. (**a**) Illustration of path. (**b**) Image of the scene.

We collect a dedicated data sequence for this calibration, where we try to excite all axes of the alignment as much as possible. The path is shown in Fig. 6. The most difficult axes to excite are roll and pitch, as the ferry prototype is a relatively stiff boat. Eight people moved in synchrony to induce roll and pitch motion in the vessel. The trajectory was also planned to pass near a low floating dock and a bridge, providing horizontal features to better constrain the LIO elevation, as most environmental structures are vertical.

A limitation encountered was that the INS/GNSS did not reliably estimate roll and pitch. This affected the estimation of the vertical z-axis(height) component of the transformation. To address this, we measured the vertical offset manually using a laser rangefinder and kept it fixed during optimization.

#### Polarized Stereo Rig Calibration

We calibrate the polarized sensor rig by first estimating the intrinsics of each of the two polarized cameras, then determining the transformation between them, and finally estimating the transformation between one of the polarized cameras and one of the other stereo cameras.

Unlike the other stereo cameras, however, the polarized stereo rig does not come with manufacturer provided intrinsic and extrinsic parameters. We do this calibration ourselves from the first calibration sequence using the calibration board shown in Fig. 4. The accuracy of the results is difficult to assess, which is why we also published the calibration sequences. They allow future work to apply improved techniques and potentially refine these results.

The extrinsic parameters, the transformations, between the two polarized cameras are estimated in the same manner as for the stereo cameras. The same approach is also applied to estimate the transformation between the polarized camera and one of the stereo cameras.

## Data Records

The dataset is published to NIRD Research Data Archive (NIRD RDA), both the full dataset^47^ and a single sequence^48^. We recommend reading in the code repository^49^ for a detailed description of how to access the datasets.

**Fig. 7:**
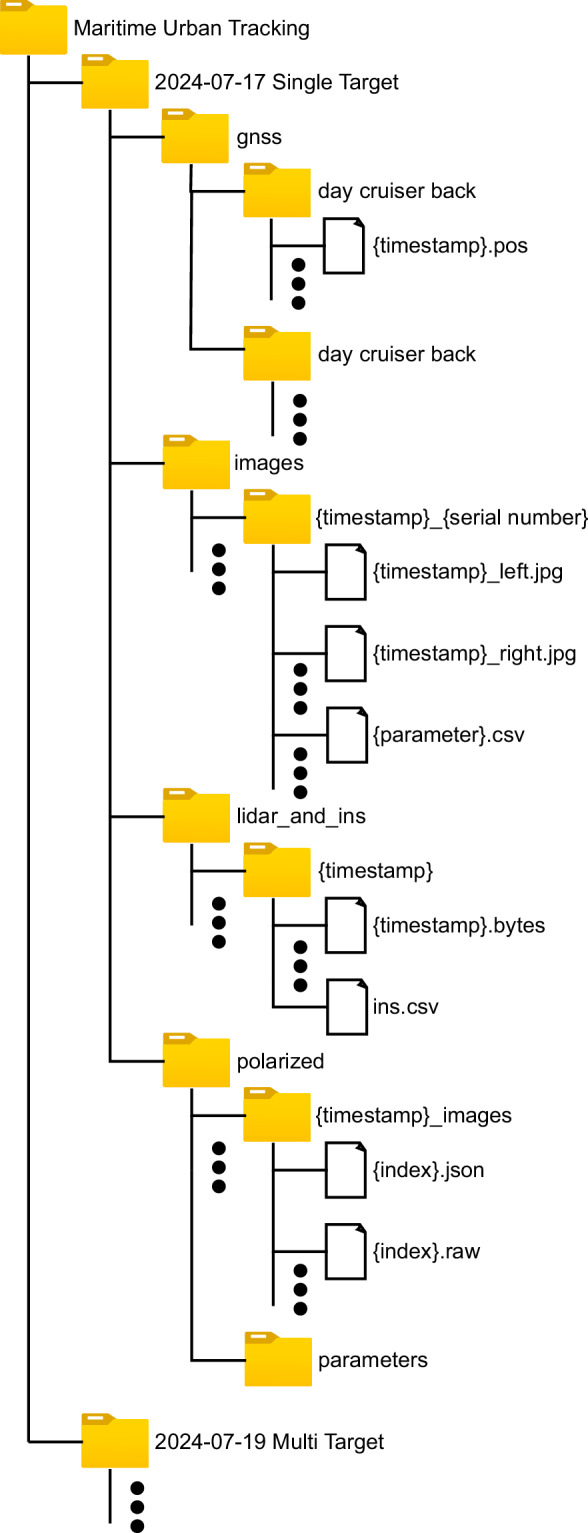
The file structure of the dataset.

The dataset is organized according to the file structure shown in Fig. 7. It is firstly split into the two days the data was gathered during: July 17 and July 19, 2024. On the first day, recordings featured a single target vessel equipped with two GNSS receivers, enabling heading estimation. On the second day two target vessels were recorded each equipped with a single GNSS receiver. Figures 10 and 11 provide an overview of the recorded trajectories and Table 6 summarizes the content of each sequence.

We have already stated in the Methods section that the recoding was using H.265 compression for the dual stereo camera setup. This was done using their proprietary software and files. Each sequence was then stored as a single file. However, for the storing of the public dataset, the images from the commercial stereo cameras are stored as “jpg” images instead of proprietary files, so that there is no need for a Graphics Processing Unit (GPU) nor their proprietary software when using the dataset. Furthermore, the images are stored unrectified and using 8 bits per channel.

The polarized data are stored as a “.bytes” file. To save space, the data is stored as bytes, although the data should be 12 bits for each cell. It is stored in such a way that 3 bytes correspond to 2 cells, like in Table 2. More details about the polarized rig and the data is described in Martens *et al*.^40^ or see the PolarizedRig class in the code.

**Table 2:** How the 12 bit data for each cell is packed into bytes to save space.

8 bit	4 bit	4 bit	8 bit
lsb	msb	lsb	msb
16 bit		16 bit	

3 bytes corresponding to 2 cells. Goes from being as in the first two rows to become as in the last row. Transformed to 16 bit unsigned integers.

All other sensor data are also provided in widely supported formats to ensure accessibility. A description of the structure of the different files types is given in Table 3.

**Table 3:** Description of the structure of the different file types used.

File type	Location	Fields	Units	Coordinate frame	Example	Timestamp / join key
.pos	gnss/**/	date, time, latitude(deg), longitude(deg), height(m), Q, ns, sdn(m), sde(m), sdu(m), sdne(m), sdeu(m), sdun(m), age(s), ratio	as named in columns	Geodetic WGS84 input; converted by code to local PIREN NED	2024/07/17 10:21:37.200 63.4342 10.3921 34.19 1 15 ...	code parses date+time to Unix ns, applies leap-second offset, then nearest-timestamp join to other sensors
.jpg	images/**/	filename-encoded timestamp_ns and side (_left / _right), JPEG payload	ns, side enum, 8-bit/ch	camera optical frames (left/right)	1721211646279279000_left.jpg	the timestamps are on trigger time, Unix ns; StereoCamera uses exact same timestamp_ns for left-right pairing; cross-sensor alignment is nearest timestamp
.csv (camera calibration)	images/**/,polarized/ parameters/	K_*.csv/K_*.csv: 3 × 3 intrinsics; D_*.csv/dist_*.csv: distortion coeffs; R.csv: 3 × 3 rotation; t.csv/T.csv: translation; optional E.csv, F.csv	pixels for intrinsics principal/focal terms, meters for translation, otherwise unitless	right -> left stereo extrinsics for R + t/T	K_left.csv: 731.89,0,939.15 / 0,731.89,511.22 / 0,0,1	static metadata in same folder as image stream
.csv (ins.csv)	lidar_and_ins/**/ ins.csv	tab-separated timestamp_ns, n, e, d, qx, qy, qz, qw	ns, m, unit quaternion	PIREN NED	1721208033499757568, 1.23, -0.45, 0.67, 0.00, 0.00, 0.00, 1.00	timestamp is direct key for nearest join with Lidar/cameras/GNSS; timestamp is always Unix ns and here it is also in trigger time
.bytes	lidar_and_ins/**/	packed PointCloud2-like records, point_step=48, fields at byte offsets: x(0,float32), y(4,float32), z(8,float32), intensity(16,float32), t(20,uint32), reflectivity(24,uint16), ring(26,uint16), ambient(28,uint16), range(32,uint32)	m for xyz; other fields are sensor-native numeric channels	lidar_aft/os_sensor (from embedded metadata)	1721207717241221888.bytes	file stem is scan timestamp_ns; code currently returns t,x,y,z per point for downstream timing compensation; t is trigger time addition to the first trigger time that is in the file stem in Unix ns
.json	polarized/*_images/*.json	top-level array with exactly two camera records; each record contains frame_id, timestamp_ns, cam_serial, Gain, ExposureTime, balance_ratio.(Red,Green,Blue), incomplete	ns for timestamp_ns, us for ExposureTime, others sensor-native	polarized left/right cameras	two-record JSON array, e.g., [record_0, record_1]	key used by code is timestamp_mean = int((timestamp_ns[0] + timestamp_ns[1]) / 2); this maps to .raw by filename stem; each of these timestamps were the trigger Unix ns time for each of the polarized cameras in the polarized sensor rig
.raw	polarized/*_images/*.raw	packed 12-bit Bayer payload, decoded in code to a (4096, 2448) array before splitting/processing	12-bit digital levels	polarized rig raw sensor grid (later rectified to left/right outputs)	000001.raw	joined to .json by identical filename stem; timestamp comes indirectly from paired .json

For the most precise details we advice the reader to read the code, see section Code Availability.

In the code we use the notation “H_points_stereo_rl_from_polarized_left” for transforms. This is the homogeneous transformation of a point in the reference frame of the left polarized camera optical frame to become a point described in the left camera on the right stereo camera. In mathematical terms it is:

$${p}^{srl}={H}_{pl}^{srl}{p}^{pl},$$

where

- *p*^*srl*^ is a point described relative to the frame of the left camera in the right stereo camera on the dual stereo camera setup.
- ${H}_{pl}^{srl}$ is the homogeneous transform.
- *p*^*pl*^ is a point described relative to the frame of the left polarized stereo camera.

Examples of coordinate frames cane be seen in Fig. 3, where the x-axis is red, the y-axis is green and the z-axis is blue. They follow theses conventions:

- The coordinate frames of the cameras have x-axis to the right, y-axis down and z-axis forward.
- The coordinate frame of the Lidar has the z-axis point upward.
- The “piren” frame is a north-east-down frame.
- The “vessel” frame as x-axis forward, y-axis to the port side and the z-axis upward. However, note that we placed the sensors on the aft of the ferry prototype. This detail should not matter for the use of the dataset.

## Data Overview

This section describes the environment in which the recordings took place. Furthermore, we give an overview of the resulting data, including scenarios, an overview of the calibration and explaining the reference tracks, see Figs. 10, 11 and Table 6.

### The Inshore Scene

**Fig. 8:**
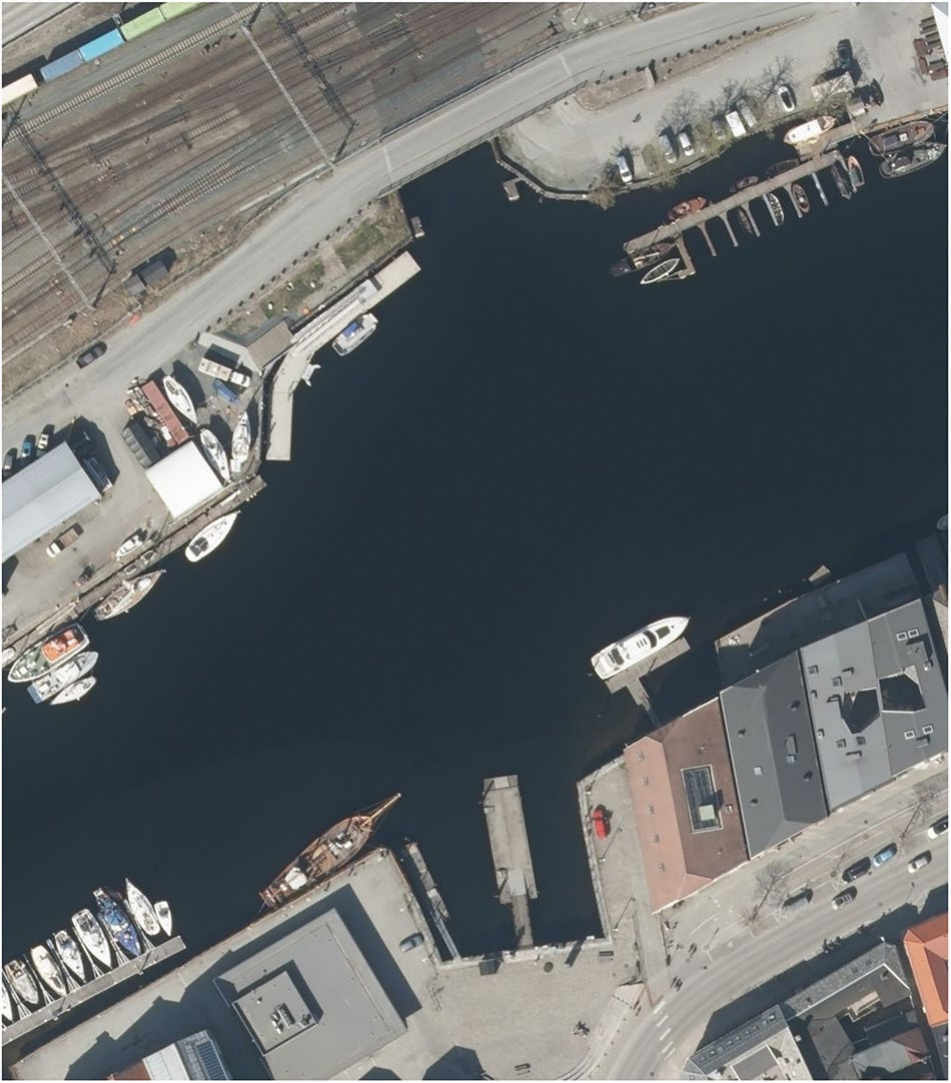
A plane photo from the scene where the dataset was recorded. ©Kartverket. Image license: CC BY 4.0.

The data was recorded in the canal between Brattøra and Trondheim city centre, Norway, as depicted in Fig. 8. Weather conditions were stable (cloudy or sunny without rainfall). The canal width ranges from 70 to 100 meters. The canal area is sheltered from the open sea and therefore largely free of significant waves, except for vessel-induced wakes. All scenarios except one, were recorded within the area shown in Fig. 8. In the mapping scenario, the ego-vessel is moving further down the canal to map a larger area. The canal has two docking points for the ego-vessel, both visible in Fig. 8, one in the upper middle section of the image and one in the lower middle section.

### Calibration

Calibration results are included in the dataset. To enable reproducibility and potential improvement, we release the raw calibration sequences alongside the dataset. Researchers may refine the calibration using alternative methods or perform joint/auto calibration across all six cameras. For reference, we provide manual measurements of the sensor rig from a tape measure in Table 4.

**Table 4:** Manual measurements.

Parameter	Measurement
Single target day 2024-07-17	
Distance between polarized cameras	95 cm
Distance between the two left cameras on the two short baseline stereo cameras	1.63 m
Distance between the right polarized camera and the left camera on the right short baseline stereo camera	30 cm
Multi target day 2024-07-19	
Distance between the two left cameras on the two short baseline stereo cameras	1.53 m
Distance between the right polarized camera and the left camera on the right short baseline stereo camera	23 cm
Distance between the left polarized camera and the left camera on the left short baseline stereo camera	35 cm

**Fig. 9:**
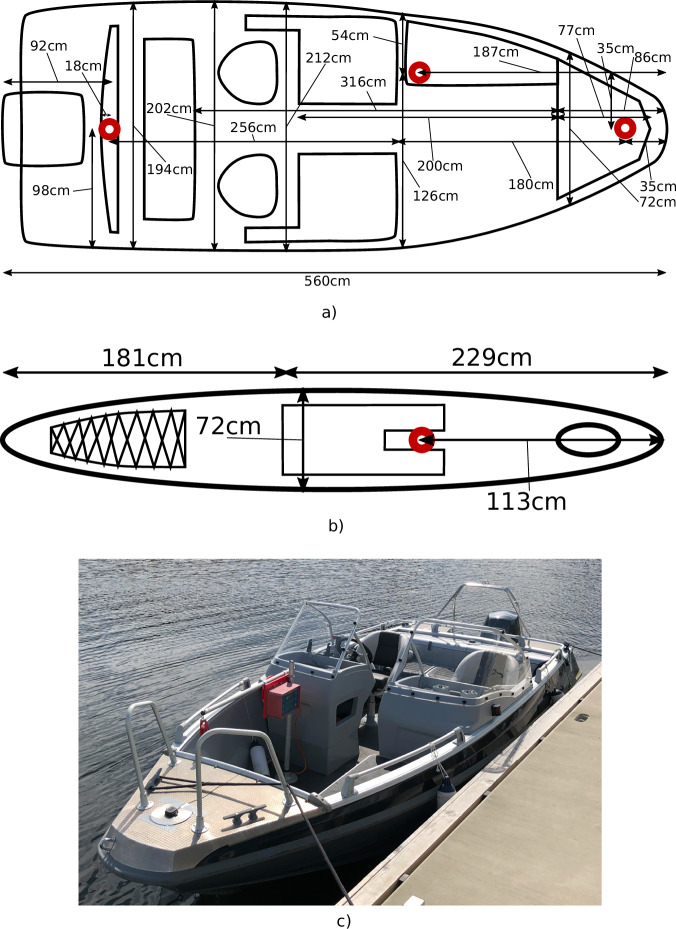
The measurements taken on the day cruiser target and the kayak. This is useful for extent ground-truth and to specify where the GNSS antennas are placed. (**a**) Measurement sketch for the day cruiser. The antenna placements are visualized in red. (**b**) Measurement sketch for the kayak. The GNSS antenna placement is visualized in red. The kayak front is on the right. (**c**) Image of day cruiser target.

These are useful to compare calibration with. The GNSS antenna placements and extent measurements are shown in Fig. 9.

The extent measurements of the targets are documented in Fig. 9. For the day cruiser, the single target day has the GNSS antennas placed one in front and one in the back. On the multi target day the single GNSS antenna is placed in the middle front of the target. The sketch is made by using an up-to-scale drawing of the vessel from its documentation. Then the manual tape measure measurements are drawn on top. These measurements allow for creating a reference extent for Extended Object Tracking (EOT)^31^.

### Reference Tracks

**Fig. 10:**
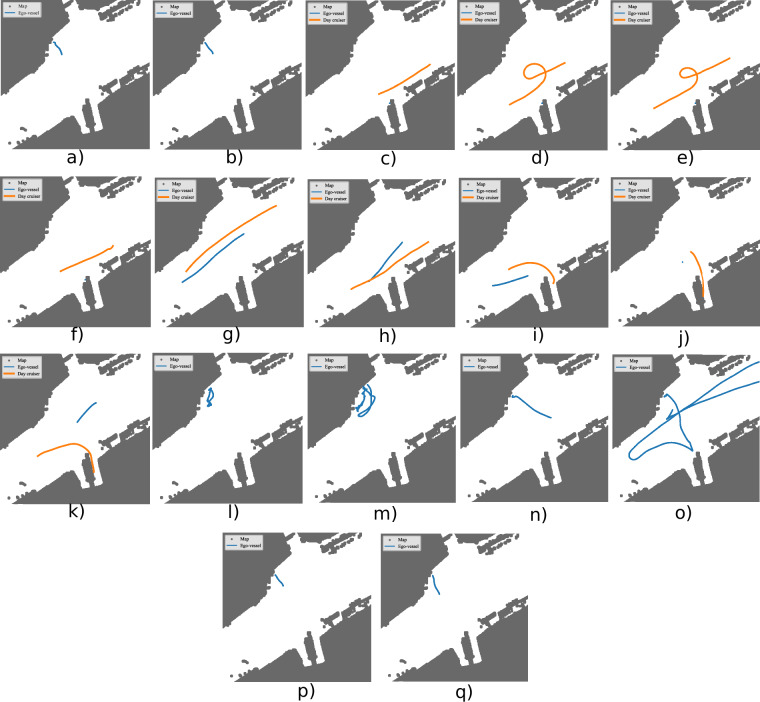
All sequences gathered on the single target day with the GNSS and INS/GNSS data plotted. (**a**) Stereo calibration 1. (**b**) Camera LiDAR calibration 1. (**c**) West 1. (**d**) Maneuver 1. (**e**) Maneuver 2. (**f**) West 2. (**g**) Pass north. (**h**) Pass south. (**i**) Undock south. (**j**) Undock still 2. (**k**) Cross. (**l**) 180 1. (**m**) 180 2. (**n**) Docking. (**o**) Mapping. (**p**) Camera LiDAR calibration 2. (**q**) Stereo calibration 2.

Target tracking is a primary application of this dataset. The GNSS receivers mounted on the target vessels enable accurate reference track generation. While the dataset does not prescribe a specific reference-track generation method, we have previously demonstrated how dual GNSS receivers can be combined to estimate both trajectory and heading^31^. Example reference tracks derived in this manner are illustrated in Fig. 10. The front GNSS and back GNSS antennas correspond to specific locations on the extended target day cruiser shown in Fig. 9. Their measurements are used in a smoothing approach, together with the manual measurements of where the antennas are placed on the vessel, to estimate the location of a specific point on the vessel and the vessel’s heading. The result is that the dataset allows not only for target tracking, but also EOT with heading reference.

**Fig. 11:**
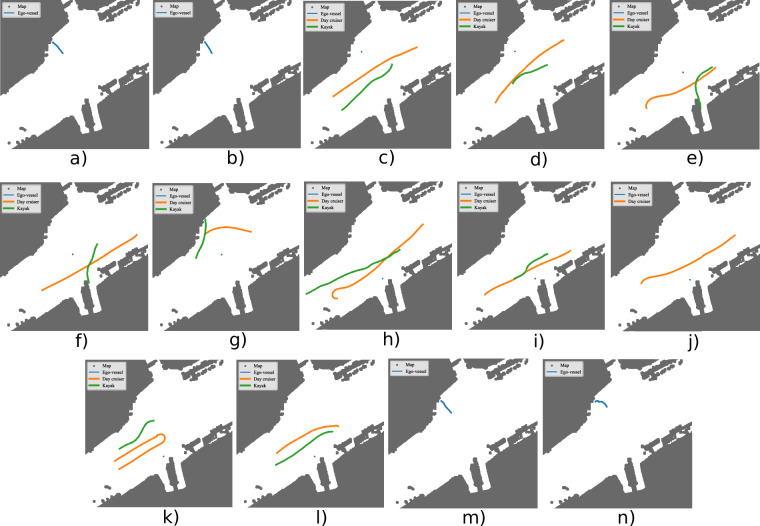
All sequences gathered on the multi target day with the GNSS and INS/GNSS data plotted. (**a**) Stereo calibration 1. (**b**) Camera LiDAR calibration 1. (**c**) Pass 1. (**d**) Pass 2. (**e**) Kayak undock 1. (**f**) Kayak undock 2. (**g**) Day cruiser undock. (**h**) Pass 3. (**i**) Pass 4. (**j**) Pass 5. (**k**) Maneuver 2. (**l**) Cross. (**m**) Camera LiDAR calibration 2. (**n**) Stereo calibration 2.

## Technical Validation

The dataset was validated quantitatively using tracking-based, calibration-based, and synchronization-based metrics. Tracking performance was evaluated by comparing estimated trajectories from multiple methods against GNSS reference tracks^30,31^, yielding a Root Mean Square Error (RMSE) of 0.21 m in north direction, 0.19 m in east direction, 7.9° in heading, $0.28\frac{{\rm{m}}}{{\rm{s}}}$ in north velocity, $0.34\frac{{\rm{m}}}{{\rm{s}}}$ in east velocity and $3.93\,\frac{\circ }{{\rm{s}}}$ in heading velocity. See tracking details in Dalhaug *et al*.^31^.

Calibration accuracy can firstly be evaluated through comparing with manual measurements. This is done in Table 5. We note that the accuracy of our measurements are likely way below the accuracy of the calibration. Calibration accuracy was also evaluated using reprojection error, with an RMSE of 0.11 px for the single target day calibration and 0.28 px for the multi target day calibration as reported by OpenCV. This evaluation used both stereo calibration sequences from each day.

**Table 5:** Validation of calibration through measuring certain distances. The manual measurements were taken with a tape measure.

Day	From	To	Meas [m]	Calib [m]	Diff [m]
Single target day	SLL	SRL	1.63	1.638	0.008
Single target day	SRL	PR	0.30	0.298	0.002
Single target day	PL	PR	0.95	0.951	0.001
Multi target day	SLL	SRL	1.53	1.539	0.009
Multi target day	SRL	PR	0.23	0.250	0.02
Multi target day	PL	PR	0.95	0.951	0.001

"SRL” means the left camera on the right stereo camera. “PR” means the right polarized camera. “Meas” means the measured distance between the sensors. “Calib” means the distance that the calibrated sensor extrinsics result in. The measured data corresponds to the data in Table 4.

**Table 6:** A description of each of the sequences taken during the single target day, see Fig. 10, and the multi target day, see Fig. 11.

Name	Start time	End time	Description
Single target stereo calibration 1	11:15	11:21	Stereo camera extrinsics calibration.
Single target camera LiDAR calibration 1	11:28	11:32	Camera LiDAR calibration.
Single target west 1	12:16	12:18	The day cruiser goes west while ego-vessel is at Ravnkloa.
Single target maneuver 1	12:21	12:22	The day cruiser moves from west and does a loop.
Single target maneuver 2	12:25	12:26	The day cruiser moves from west in a loop again.
Single target west 2	12:28	12:29	The day cruiser moves from east in front of the ego-vessel that is docked at Ravnkloa.
Single target pass north	12:32	12:25	The ego-vessel moves from west and the day cruiser moves from east. The ego-vessel passes south of the day cruiser.
Single target pass south	12:38	12:39	The day cruiser moves from west while the ego-vessel moves from east. The day cruiser passes south of the ego-vessel.
Single target undock south	12:47	12:49	The day cruiser undocks at Ravnkloa while the ego-vessel moves from west in front.
Single target undock still 2	13:41	13:42	The ego-vessel stands still while the day cruiser is undocking at Ravnkloa.
Single target cross	13:50	13:51	The ego-vessel moves slowly forward while the day cruiser crosses in front.
Single target 180 1	13:57	14:02	180^°^ view of dock.
Single target 180 2	14:40	14:49	180^°^ view of dock again.
Single target docking	14:55	14:58	The ego-vessel docks.
Single target mapping	15:06	15:21	We map the region by moving the ego-vessel along the canal, first east, then west. Then we get close to the dock at Ravnkloa before we move north to dock at Brattøra.
Single target camera LiDAR calibration 2	15:27	15:31	Camera LiDAR calibration.
Single target stereo calibration 2	15:34	15:39	Stere camera extrinsic calibration.
Multi target stereo calibration 1	09:11	09:16	Stereo camera extrinsics calibration.
Multi target camera LiDAR calibration 1	09:20	09:24	Camera LiDAR calibration.
Multi target pass 1	09:37	09:39	The day cruiser and kayak pass each other.
Multi target pass 2	09:40	09:41	The day cruiser and Kayak pass each other again on opposite side.
Multi target kayak undock 1	09:44	09:45	The day cruiser moves in front of dock and the kayak undocks from behind.
Multi target kayak undock 2	09:47	09:49	The day cruiser moves from the other side and crosses dock while the kayak undocks from behind.
Multi target day cruiser undock	09:54	09:55	The day cruiser undocks from Brattøra and the kayak comes from behind going west.
Multi target pass 3	10:09	10:11	The ego-vessel looks north while the day cruiser comes from east and the kayak comes from west.
Multi target pass 4	10:11	10:12	The ego-vessel looks north while the day cruiser comes from west and the kayak comes from east.
Multi target pass 5	10:13	10:15	The ego-vessel looks north while the day cruiser comes from east and the kayak comes from west. With a boat in the background.
Multi target maneuver 2	10:23	10:25	The day cruiser does loop from west while the kayak is in the background.
Multi target cross	10:27	10:29	The kayak moves from west and the day cruiser moves from east, crossing each other.
Multi target camera LiDAR calibration 2	10:40	10:43	Camera LiDAR calibration.
Multi target stereo calibration 2	10:44	10:45	Stereo camera extrinsics calibration.

Ravnkloa is the southern dock while Brattøra is the northern dock.

Synchronization accuracy was assessed via manual real world event detection in the different sensor data. Taking the left stereo camera as a reference, the right stereo camera got a time difference of 0.063 ms, the polarized rig got 12.0 ms and the Lidar got 68.3 ms. Note that the two stereo cameras should have very similar trigger times. Furthermore, the uncertainty of this is limited to selecting the correct frames and therefore depends on the frame rate of each sensor and the temporal alignment of the triggering.

Visual inspection of all sequences was additionally performed to verify the absence of systematic misalignment not captured by aggregate metrics.

## Data Availability

The dataset can be found at NIRD Research Data Archive (NIRD RDA)^47^. We recommend that users start by reviewing the code examples provided in the code repository (see the next section) before exploring the data files. We have also published a single scenario^48^ of the dataset to make it easier to try out the dataset without downloading the full version.

## Code availability

To simplify use, we provide a Python library for reading and processing the dataset^49^. How each file maps to each scenario is listed with easy-to-use abstractions for basic data reading. Users may extend the library for advanced applications or use it as a reference for implementing their own readers.

In addition, code is provided to perform demosaicking of the raw color polarization video from the sensor rig, using the method and weights described in Martens *et al*.^40^. This method simultaneously performs color and polarization demosaicking, using a single convolution operation^40^. It yields better results with fewer aliasing artifacts than using regular debayering on each polarization channel individually, as it takes the physical filter offsets into account^40^.
